# Enhancing health system governance and citizen empowerment in Bhutan through the Bhutan Vaccine System (BVS): a mixed-methods study

**DOI:** 10.3389/fpubh.2026.1828261

**Published:** 2026-07-01

**Authors:** Sonam Yangchen, Tashi Dawa, Cheten Zangmo, Dechen Tshomo, Mongal Singh Gurung

**Affiliations:** 1 Institute of Health Partners, Thimphu, Bhutan; 2 Ministry of Health, Royal Government of Bhutan, Thimphu, Bhutan; 3 Royal Thimphu College, Thimphu, Bhutan

**Keywords:** Bhutan, citizen empowerment, COVID-19 vaccination, digital health, governance, health-systems strengthening, immunisation information system

## Abstract

**Background:**

The Bhutan Vaccine System (BVS) was developed for the COVID-19 vaccination and later expanded to include influenza and HPV vaccinations. It represents a key national innovation in health system transformation, linking vaccination delivery with citizen engagement. This study assessed how the BVS strengthened vaccination monitoring, governance processes, and citizen empowerment in Bhutan.

**Methods:**

We used a convergent mixed-methods design integrating secondary analysis of BVS data with qualitative interviews. Quantitative data from the BVS were analysed by age, sex, and district. Qualitative data were drawn from 56 key informants comprising policymakers, health workers, and community members from six districts of Bhutan. Thematic analysis, guided by Potter and Brough’s systemic capacity-building framework, identified enablers and barriers across performance, personal, workload, facility, and support capacities. Findings were integrated using a convergence framework to align quantitative trends with qualitative themes.

**Results:**

By December 2023, 91.6% of Bhutan’s total population had received at least one COVID-19 vaccine dose, and 83.3% had received three or more doses. Policymakers and health workers reported that the BVS improved real-time monitoring, reduced administrative delays, and data visibility. Self-registration and downloadable digital certificate features of the BVS increased people’s autonomy and trust. Key barriers included limited interoperability with other health-information systems, unreliable internet connectivity, reporting burdens, and insufficient user training and hardware.

**Conclusion:**

The BVS demonstrates how a digital platform can strengthen governance and empower citizens when integrated within primary health-care structures. Sustained investment in infrastructure, digital literacy, and interoperability will be critical to maintain these gains and to guide equitable digital-health governance across low and middle-income countries. Bhutan’s experience offers practical lessons on how digital systems can advance both efficiency and equity in public health management.

## Background

Digital innovations have been adopted by health systems worldwide to advance the 2030 Sustainable Development Goals (SDGs), particularly SDG 3 (health and well-being). Among others, many countries have introduced electronic immunization information systems to improve coverage, monitor vaccine logistics, and enhance accountability (1–4). Evidence indicates that, when well designed and contextually adapted, such systems can strengthen service delivery, transparency, accountability, and citizen participation (5–10).

Bhutan, a small Himalayan country with a 2023 population of 770,276, provides free universal health care as mandated by its Constitution. Despite this commitment, the health system continues to face challenges arising from rugged terrain, dispersed settlements, and a limited workforce. There are approximately 12 health professionals per 10,000 population in Bhutan which is only half the WHO benchmark of 23 (4). Delivering equitable services to remote areas therefore remains resource-intensive and logistically demanding. At the same time, the country’s small size presents an opportunity to design and implement nationwide interventions. Over the past decade, Bhutan has pursued rapid digital transformation guided by the National Digital Health Strategy. Core digital platforms include the District Health Information System (DHIS2) for routine health data, the Electronic Patient Information System (ePIS) for facility-level clinical records and medical supply inventory, the National Early Warning Alert and Response Surveillance (NEWARS) system for communicable-disease surveillance, and the Bhutan Vaccine System (BVS) for COVID-19. Other routine under-five vaccinations are recorded through the ePIS platform.

The BVS is a national web-based platform that enables digital registration, consent management, logistics tracking, real-time monitoring through dashboards accessible at all administrative levels, and Adverse Event Following Immunization (AEFI) reporting by both citizens and health-care providers. Each individual registered in the BVS was assigned a unique registration number linked to the national Citizenship Identity (CID) system to support identity verification, deduplication, and longitudinal tracking. Building on its success, the Ministry of Health subsequently expanded the BVS to include two additional routine vaccines (influenza and HPV) thereby extending the platform beyond COVID-19 vaccination.

While immunisation information systems are increasingly common in low and middle-income countries (LMICs), relatively few studies have explored their broader governance implications, including how they may influence accountability between governments and citizens. In Bhutan, where public trust and community participation underpin health service delivery, the BVS offers a distinctive opportunity to examine how digital vaccination platforms may influence governance processes and citizen engagement within an LMIC context.

This study aimed to (i) analyse national vaccination coverage patterns using data from the Bhutan Vaccine System (BVS), and (ii) examine how BVS contributed to health system governance—particularly accountability, transparency, and citizen engagement—through stakeholder perspectives using a convergent mixed-methods approach.

## Methods

### Study design

We used a convergent mixed-methods design, in which the quantitative component utilised routinely collected BVS data recorded in real time during the vaccination programme, while qualitative data were collected during October–November 2023. Both components were analysed independently and integrated at the interpretation stage. This approach enabled triangulation of system-generated evidence with perspectives from key stakeholders—including policymakers, health workers, and community members—providing a comprehensive understanding of the BVS as both a technological and governance innovation.

### Study setting

The study was conducted in Bhutan, a lower–middle-income country situated in the eastern Himalayas between India and China. Health services are delivered through a three-tier system: (i) national and regional referral hospitals at the tertiary level, (ii) cluster and general hospitals at the secondary level, and (iii) an extensive network of Primary Health Centres (PHCs), Thromde Health Centres (THCs), and sub-posts at the primary level. PHCs, staffed mainly by health assistants who also provide midwifery and maternal and child health services, constitute the main point of care for most rural populations, including those living in remote mountain settlements. Traditional Medicine is also provided from the same hospitals under an integrated health system.

### COVID-19 vaccination programme context

Bhutan’s COVID-19 vaccination programme was implemented in alignment with global regulatory approvals and national risk stratification. Vaccines were initially authorised for adults aged 18 years and above, who received up to four doses. Following subsequent international and national approvals, eligibility expanded to adolescents aged 12–17 years and later to children aged 5–11 years, who received up to three doses in accordance with national guidelines. A fifth booster dose was offered to high-risk groups, particularly older adults (≥65 years) and individuals with comorbidities, in line with recommendations from the National Immunization Technical Advisory Group (NITAG) and the WHO Strategic Advisory Group of Experts on Immunization (SAGE).

Multiple vaccine types were used during the campaign, including AstraZeneca, Covishield, Moderna, Pfizer, and Sinopharm. The first two doses were administered using either homologous or heterologous combinations depending on availability and individual choice, with AstraZeneca–Moderna being the most common regimen (over 93% of recipients). The bivalent Pfizer formulation was later introduced for the fifth booster dose targeting high-risk groups.

### Participants

#### Quantitative component

The quantitative component utilised national-level BVS data representing a near-complete operational registry of individuals registered during the vaccination campaign. Vaccination coverage estimates were calculated using projected target population denominators published by the National Statistics Bureau of Bhutan rather than the number of individuals registered in the BVS. Numerators were derived from vaccination records contained within the BVS registry. By December 2023, a total of 731,562 individuals were registered in the BVS, representing approximately 95% of Bhutan’s 2023 population of 770,276 (11). The dataset was extracted from the central BVS database, which records all individuals registered for vaccination across all districts and population groups aged 5 years and above. Variables included age, sex, vaccination status (dose number, vaccine type, and place of vaccination), comorbidities, and place of residence.

Data were subjected to routine quality checks, including removal of duplicate records and verification of key variables. Missing data were minimal, with less than 0.02% missingness for age and 0.04% for district, while no missing data were observed for sex or vaccination dose variables. Records with missing values were excluded from subgroup analyses.

Given the population-level nature of the dataset, the analysis focused on descriptive and comparative statistics, including proportions and stratified distributions by age, sex, and geographic region. Temporal patterns across vaccination phases were also examined to assess programme dynamics.

#### Qualitative component

In-depth interviews were conducted with purposively selected stakeholders involved in the design, implementation, and use of the Bhutan Vaccine System (BVS). A total of 56 participants were recruited from seven districts representing Bhutan’s western, central, and eastern regions to capture contextual and geographic diversity. Two districts per region were selected from eastern and central region in consultation with the Ministry of Health and district authorities and three districts were selected from western Bhutan. From each district one rural healthcare facility and one urban health facility were purposively selected. At each selected health facility one health worker and two community members were interviewed.

Participants were grouped into three stakeholder categories:

(i) Policymakers and programme managers (*n* = 14), including officials from the Policy and Planning Division, Health Management Information System Unit, Vaccine-Preventable Diseases Programme, and district health offices;
(ii) Health workers (*n* = 14), including PHC in-charges, Maternal and Child Health in-charges in hospitals, and medical-record officers who directly used the BVS; and
(iii) Community members (*n* = 28), including village leaders, community representatives, and residents from rural and urban areas who interacted with the BVS for registration, consent, or vaccination.

This sampling strategy enabled triangulation across national, facility, and community levels, with maximum variation ensuring inclusion of both urban and remote rural settings.

#### Data collection

Qualitative data collection took place from October to November 2023, and the quantitative dataset reflected records up to 31 December 2023. Qualitative data were obtained through in-depth interviews conducted in Dzongkha, English, or local languages, depending on participant preference, using interview guides prepared by the study team. Interviews were held privately, audio-recorded with informed consent, and transcribed verbatim. Interviews lasted approximately 30–60 min and followed a standardised guide (Supplementary Annex S1).

#### Ethical considerations

Prior to data collection, administrative clearance was obtained from the Ministry of Health (MoH/PPD/ADM. CL/2023/042), and ethical approval was granted by the Research Ethics Board of Health (REBH/Approval/2023/026). While the REBH waived the need for informed consent for the BVS dataset, the participants of qualitative interviews provided written informed consent. Interview transcripts and quantitative data from the BVS were pseudonymised and stored on password-protected computers accessible only to the research team. No personal identifiers were used in the analysis or reporting.

### Quantitative analysis

The BVS dataset was exported as CSV files and analysed using Stata version 18. Because the dataset represented a near-complete operational registry of individuals registered in the national vaccination programme, unweighted analysis was performed, and results are presented as descriptive comparisons. Descriptive statistics summarised vaccination coverage by age, sex, and district, and trends were visualised using bar charts and cross-tabulations. Given that the BVS registry captured approximately 95% of the projected national population rather than a sampled subset, inferential statistical testing was not considered necessary, and emphasis was placed on descriptive and stratified analyses to characterise population-level patterns.

### Qualitative analysis

The qualitative data from the in-depth interviews (IDIs) were transcribed and analysed using a thematic framework approach. Each interview was audio-recorded with consent and transcribed verbatim by the research team. The transcripts were read several times by two researchers to familiarise themselves with the content before being imported into webQDA for coding and analysis. A hybrid inductive–deductive approach was applied, allowing codes to emerge from the data while also being informed by the Potter and Brough systemic capacity-building framework (8). This framework guided the categorisation of findings under five capacity domains: performance, personal, workload, facility, and support. Two researchers independently coded a subset of transcripts to ensure consistency, and discrepancies were resolved through discussion. Emerging subthemes were iteratively reviewed against the framework to maintain analytic rigour and to interpret findings in the context of the study objectives.

### Integration of findings

Integration was guided by a convergence framework that aligned quantitative trends with qualitative themes. Quantitative and qualitative findings were first analysed independently and then integrated at the interpretation stage using a convergence framework. Quantitative vaccination coverage patterns were systematically compared with qualitative themes derived from stakeholder interviews to identify areas of convergence, complementarity, and divergence. Qualitative findings were used to contextualise and explain observed quantitative trends, particularly regarding governance processes, implementation experiences, and citizen engagement. Integration was further reflected through linking statements within the Results section connecting quantitative findings with qualitative insights.

## Results

### Secondary analysis of Bhutan vaccine system data

During the initial phase of the campaign, vaccination coverage exceeded 90% across all eligible population groups, including adults, adolescents, and children eligible for vaccination during the respective phases of the national COVID-19 vaccination programme. The vaccination coverage was sustained over time, supported by continuous advocacy and real-time monitoring through the BVS dashboard. This high level of coverage is consistent with qualitative findings highlighting the role of real-time monitoring and central dashboards in enabling rapid identification and follow-up of unvaccinated individuals. Participants also described how BVS data were cross-checked against census-based population estimates, local government records, and health facility population records collected through the Annual Household Health Surveillance system to help identify potentially unreached or unregistered individuals.

By December 2023, 91.6% of Bhutan’s total population had received at least one dose, 88.7% had received at least two doses, and 83.3% had received at least three doses (Table 1). Coverage for the fourth and fifth doses reached 55.1 and 6.9%, respectively. Among individuals aged 5 years and above, uptake was even higher, with 99.3% receiving the first dose, 96.1% the second dose, and 90.4% the third dose.

**Table 1:** COVID-19 vaccination coverage by dose and background characteristics, Bhutan, December 2023 (Bhutan vaccine system dataset).

Background characteristics	Percentage of population who received various COVID-19 doses					Number of population
	Dose 1	Dose 2	Dose 3	Dose 4	Dose 5	
Age group (in years)						
5–11	100.0	100.0	89.2	2.2	0.0	81,375
12–17	100.0	100.0	99.9	58.6	1.0	78,265
18–24	100.0	100.0	93.6	65.0	1.0	95,849
25–34	92.5	90.9	84.9	58.5	1.9	151,993
35–44	90.0	88.9	86.6	67.0	4.0	122,186
45–54	94.9	93.3	92.5	75.8	7.4	77,249
55–64	88.6	87.7	87.4	74.4	13.0	51,806
65 & above	99.4	96.9	96.1	89.7	60.0	51,945
Sex						
Female	94.4	92.3	87.9	60.8	8.2	360,404
Male	94.4	90.6	84.2	53.4	6.3	391,673
District						
Bumthang	90.2	93.5	81.7	57.5	6.7	18,397
Chukha	94.7	84.2	81.2	57.0	4.2	70,020
Dagana	91.8	86.7	84.3	56.6	7.5	25,901
Gasa	79.1	81.0	63.3	55.3	9.5	4,362
Haa	85.8	87.6	77.3	57.7	5.9	13,850
Lhuentse	99.5	97.1	95.2	73.9	14.8	13,464
Mongar	98.5	96.5	95.0	68.9	10.6	36,228
Paro	92.2	90.9	83.9	55.1	6.9	51,912
Pemagatshel	91.4	88.3	87.4	69.9	12.5	23,840
Punakha	94.8	83.4	81.1	53.8	7.1	31,233
Samdrupjongkhar	98.5	93.1	90.9	63.8	10.9	35,079
Samtse	95.7	91.7	90.8	65.2	8.7	63,149
Sarpang	95.0	90.3	90.7	54.4	8.5	50,221
Thimphu	85.6	89.3	78.8	40.7	2.3	162,593
Trashigang	93.9	88.8	84.3	63.5	9.9	42,298
Trashiyangtse	98.9	95.7	95.5	65.2	12.3	16,573
Trongsa	70.6	66.3	63.3	47.0	7.1	23,150
Tsirang	91.7	88.6	82.7	59.5	8.7	24,073
Wangdue	88.7	81.3	74.8	52.7	6.0	47,377
Zhemgang	98.7	93.7	91.4	62.4	13.8	16,753
Population (5 years and above)	99.3	96.1	90.4	59.7	7.5	710,667
Population (all ages)	91.6	88.7	83.3	55.1	6.9	770,276

Percentages are calculated based on the total population in each subgroup.

**Figure 1:**
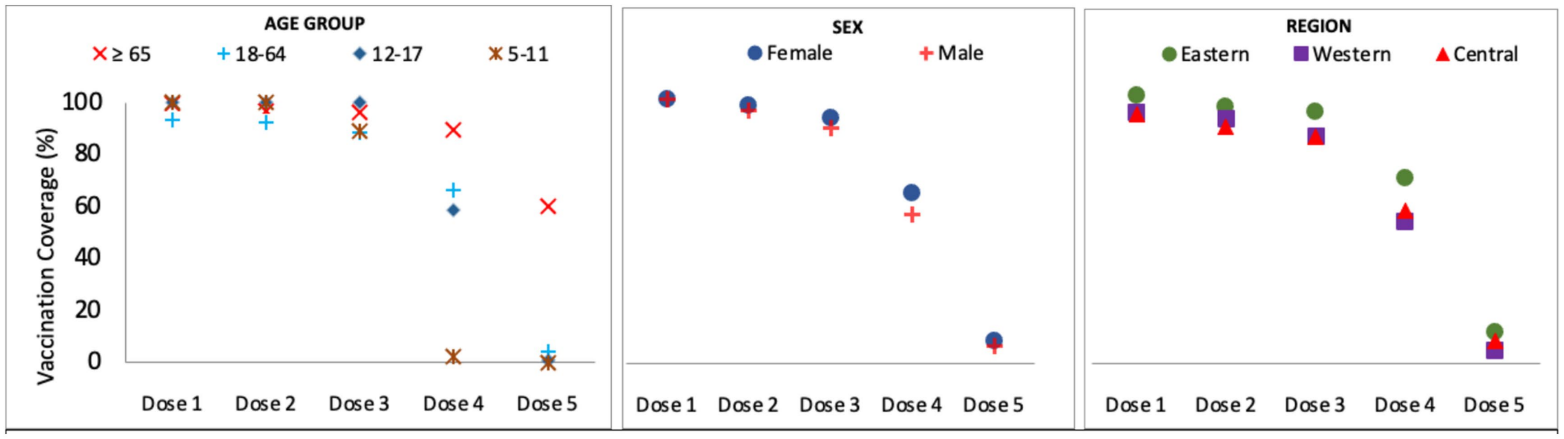
COVID-19 vaccination coverage by dose, age group, sex, and region in Bhutan, 2023. Each panel shows the percentage of the population receiving each vaccine dose (1–5), illustrating variations in booster uptake across demographic groups. Coverage (%) = (Number of individuals who received the dose/Target population for the dose) × 100. Data source: Bhutan vaccine system (BVS), 2023.

Figure 1 shows vaccination coverage by age, sex, and region. Coverage remained consistently high (>90%) for the first three doses, with higher uptake of the fourth and fifth doses among older adults. Females had slightly higher booster coverage, and the eastern region maintained marginally higher first-dose coverage than other regions. Qualitative findings further suggest that these variations reflect differences in local implementation capacity and logistical coordination.

### Qualitative findings

Six overarching themes emerged across the three respondent groups, corresponding broadly to the capacity domains in Potter and Brough’s framework (Table 2): (i) data quality and use, (ii) citizen empowerment, (iii) mobility and identification, (iv) system design and interoperability, (v) reporting burden and workload, and (vi) systemic capacity building. Policymakers and programme managers primarily emphasized governance, interoperability, and monitoring functions; health workers focused more on implementation challenges, workload, and infrastructure; while community members highlighted accessibility, trust, and user experience. Several themes spanned multiple capacity domains, particularly performance, workload, facility, and support capacities. Together, these themes illustrate how the Bhutan Vaccine System (BVS) influenced both governance processes and daily health-service operations.

**Table 2:** Thematic categories and illustrative sub-themes from qualitative analysis of the Bhutan vaccine system (BVS), based on Potter and Brough’s framework.

Category	Themes/sub-themes
1. Data quality and use (Enablers)	• Completeness and timeliness: accuracy of data entry and validation across health facilities. • Data usage: application of BVS data for planning, forecasting, and performance monitoring. • Data types: individual-level, aggregate, real-time vaccination information enabling follow-up.
2. Citizen empowerment (Enablers)	• Self-downloadable vaccination certificates: citizens’ ability to generate digital vaccination certificates independently. • Self-registration and consent: online enrolment allowing informed decision-making. • Mobile access: convenience of using BVS on smartphones, reducing dependence on health staff.
3. Mobility of individuals (Enabler)	• Unique identifier: linkage of records through citizenship ID and contact details. • Duplicate records: duplication arising from population mobility across districts.
4. System design and interoperability challenges (Barriers)	• Lack of interoperability and digital literacy: coexistence of multiple systems and variable user capacity. • Limited rights to edit: restricted authority of PHC users to correct minor data errors. • Denominator issues: inaccuracies in target population estimates due to migration.
5. Reporting burden on PHC managers (Barrier)	• Hard-copy records: continued maintenance of paper forms alongside BVS entries. • Multiple reporting mandates: overlapping requirements from DHIS2, MCH Tracker, and other programmes.
6. Gaps in systemic capacity building (Barrier)	• Tools: shortage of functional computers, tablets, and reliable internet connections. • Skills: limited practical training for health workers and data staff. • Staff and infrastructure: inadequate human resources and facility support. • Structure, systems, and roles: fragmented governance and unclear accountability for system maintenance.

Categories and themes were derived using Potter and Brough’s systemic capacity-building framework to distinguish enablers and barriers affecting BVS implementation.

### Data quality and use

Participants consistently reported that the BVS enhanced data completeness and timeliness. Health workers described routine verification of records to maintain accuracy.

“In the BVS we have contact numbers of most people, so we can trace them and correct missing data, though it takes time.”—Policymaker.

“Small errors remain, but overall it substantially improved monitoring coverage; we could cross-check with census estimates.”—Policymaker.

BVS data were also used for real-time decision-making.

“We use BVS data for vaccine forecasting; for example, we can see which age groups are more likely to opt for the flu vaccine.”—Programme manager.

“During campaigns, we watched live dashboards and pushed underperforming districts.”—Policymaker.

“It even helped travelers. They could instantly generate vaccination certificates.”—Health worker.

Participants also described how comparative dashboard monitoring encouraged informal learning and exchange between districts, particularly when lower-performing districts consulted higher-performing districts regarding mobilisation and follow-up strategies. At health facility, district, and national programme-monitoring levels, BVS registration data were routinely cross-checked with national population statistics, Annual Household Health Surveillance records, and local administrative records to triangulate denominator estimates and identify potentially unreached or unregistered individuals.

These reported uses of BVS data may help explain the high and sustained coverage observed in the quantitative analysis, particularly the rapid uptake during early phases of the vaccination campaign.

These findings primarily reflect strengthened performance capacity through improved data visibility and use for programme monitoring.

### Citizen empowerment

The BVS was widely perceived as a tool for citizen empowerment, shifting control and decision-making from health workers to the public. Self-registration, electronic consent, and online access to vaccination records gave individuals greater autonomy.

“Before, we simply followed what the health workers told us. With BVS, I could decide when and where to register.”—Community member.

“Now I can download my own certificate without going to the hospital.”—Community member

“It saves both time and cost; the same model should apply to other services like lab reports.”—Community leader.

Mobile-phone accessibility further improved convenience for the people:

“For introverted people like me, online registration is easier; I don’t have to approach anyone.”—Urban resident.

However, policymakers observed registration errors caused by limited digital literacy:

“Many people registered with wrong locations. If health workers handled registration, it would have been cleaner.”—Policymaker.

Despite these issues, community members, particularly younger participants and urban residents, viewed self-registration and online access as a significant cultural shift toward participatory governance in health service delivery in Bhutan.

This theme relates mainly to personal and performance capacity through increased public participation and engagement with digital health services.

### Mobility and identification

To address challenges resulting from high internal mobility among Bhutanese, the BVS assigns a unique registration number linked to each citizen’s national identity (CID) and phone number, which greatly improved traceability, though it did not fully eliminate duplication when individuals forgot their identifiers.

“People move often; one may get the first dose in one district and the second elsewhere. Without BVS, we would never reconcile records.”—Health worker.

“Earlier, some districts showed more than 100% coverage because people registered in one area but were vaccinated in another.”—Policymaker.

Although the BVS already utilised Citizenship Identity (CID) numbers for individual identification and deduplication, participants recommended further integration with Bhutan’s National Digital Identity (NDI) platform. Unlike the CID system, the NDI is a biometric digital identity platform that enables secure digital authentication, passwordless system access, and user-controlled sharing of digital credentials across interoperable services.

“Linking BVS with NDI will help us verify citizens instantly and avoid double registration.”—IT official.

These experiences reflect support and performance capacities related to identity verification and continuity of service access.

### System design and interoperability

Parallel information systems were seen as a major limitation. While BVS captured individual-level vaccination data, DHIS2 continued to host aggregated routine data, and the two systems were not interoperable.

“DHIS2 and BVS don’t communicate; we have to enter data twice.”—Health assistant

“Ideally, vaccine inventory and individual data should be in one place, but we depend on external IT support.”—Policymaker.

These interoperability limitations contributed to duplicated data entry requirements and reporting inefficiencies during implementation.

These findings primarily reflect limitations in facility and support capacities, particularly regarding interoperability and digital infrastructure.

### Digital literacy and usability challenges

Participants also described usability challenges related to digital literacy and technical support.

“Older staff struggle with log-ins and passwords. When the system freezes, we must call IT officials.”—Health worker.

Several respondents stressed the need for local development capacity so that programmes could adjust the system without external contractors.

“Even small edits, like correcting names, require going through IT. Some flexibility for field users would save time.”—District Health Officer.

### Reporting burden and workload

Despite digitalisation, providers still maintained paper registers as back-up records due to unreliable electricity or internet connectivity.

“During vaccination drives everyone was online, so the system slowed; we had to keep manual registers as back-ups.”—Health worker

“We were instructed to record on paper if the system was down, so we did double work.”—Health worker.

Primary-health-care managers noted competing reporting requirements across systems: BVS, DHIS2, ePIS, and NEWARS.

“At the end of the day, it’s the same health assistant punching data into four systems.”—Policymaker

“One HA in my centre spends half the week just on reporting; weekly, monthly, and annual forms.”—Health worker.

Participants viewed integration and automation as essential for sustainability.

“If BVS could generate reports for the programme automatically, we wouldn’t need to maintain separate Google sheets.”—District Health Officer.

This theme mainly reflects workload capacity challenges associated with parallel reporting systems and duplicated data entry.

### Systemic capacity building

Capacity constraints cut across all levels: tools, skills, staffing, and structural governance.

#### Tools and infrastructure

Participants described significant infrastructure constraints affecting BVS implementation, including unreliable internet connectivity, limited access to computers, and inadequate digital equipment at health facilities. These limitations affected the performance capacity required for effective implementation of the BVS.

“Our internet connection drops often, and only one desktop works. We sometimes use our own phones for data entry.”—Health worker.

“Some facilities still use obsolete computers that cannot connect to Wi-Fi.”—Policymaker

“If each staff member had a personal laptop, reporting could continue even during power cuts.”—Health worker.

#### Skills and training

According to key informants, training was provided but was not adequate.

“Online training was useful but not enough; hands-on sessions would help.”—Health worker

“After training, people still made errors because they lacked confidence using new tools.”—Policymaker.

#### Staffing and workload

Participants reported that most health facilities suffered from shortage of staff, resources, and supervisory support from Central Program.

“BHUs [Basic Health Unit, currently referred to as a Primary Healthcare Centre (PHC)] need at least three staff; two is not enough to maintain services and data entry.”—Health worker.

“Every programme wants its own system, but the same person has to handle them all in the field.”—Health worker.

#### Structure and governance

“We need clear forums for feedback; developers should consult data analysts and users during system development and before updates.”—Policymaker.

“When eligibility rules changed, we had to modify data manually because the system was static.”—Health worker.

Together, these perspectives underscored the need for comprehensive capacity development. Not only improved hardware and connectivity but also strengthened human skills, supervisory support, and institutional coordination.

Together, these findings illustrate how BVS influenced multiple capacity domains, including performance, facility, workload, support, and personal capacities within Bhutan’s health system.

## Discussion

This study contributes empirical evidence on how a national immunisation information system may influence governance processes and citizen engagement within an LMIC context. The findings suggest that the Bhutan Vaccine System (BVS) improved real-time monitoring, data visibility, and citizen engagement within the vaccination programme. These outcomes contribute to ongoing debates in Health Research Policy and Systems on how digital tools influence governance relationships within low and middle-income countries (LMICs).

Taken together, the quantitative and qualitative findings suggest that BVS influenced vaccination outcomes not only through improved data management but also by strengthening governance mechanisms. Real-time visibility of coverage data enabled accountability across administrative levels, while citizen-facing features enhanced engagement and trust. This interaction between technological functionality and governance processes appears central to the system’s effectiveness.

### Health system governance and accountability

The BVS enhanced individual-level visibility within Bhutan’s immunisation programme through real-time dashboards that enabled the Ministry of Health and district officials to monitor coverage, identify underperforming areas, and implement rapid corrective measures. Such transparency exemplifies adaptive governance, in which continuous feedback and learning inform responsive decision-making (1, 9).

The system was developed by a local private software firm with funding support from the United Nations Development Programme (UNDP), and subsequently adopted and maintained by the Ministry of Health as part of a broader ecosystem of nationally owned digital public goods. This collaborative model combined international support with domestic technical capacity, supporting rapid deployment during the pandemic while creating a foundation for continued institutional use within Bhutan’s digital health ecosystem. Comparable experiences elsewhere reinforce these observations. For example, India’s CoWIN platform and Rwanda’s Irembo e-health portal likewise demonstrated that transparent, centralised information systems can reinforce public confidence and institutional accountability (2, 5).

Through visible data and citizen access, BVS appeared to strengthen accountability, data visibility, and citizen participation within the vaccination programme (10, 12). Participants also described how comparative dashboard monitoring encouraged informal peer learning and exchange between districts during vaccination campaigns, mirroring effects seen in other performance-based governance models (13).

### Citizen empowerment and social trust

BVS reshaped interactions between citizens and the health system in vaccine delivery as self-registration, informed consent, and downloadable digital certificates gave people greater control over their health information, marking a shift from hierarchical service delivery to participatory engagement.

These results align with literature on digital empowerment, which views technology as a means of expanding individual autonomy as well as efficiency (14, 15). In Bhutan, pre-existing trust in government institutions and community solidarity facilitated acceptance of the platform. Participants described greater convenience and autonomy in accessing and managing vaccination records through the BVS.

Yet empowerment was uneven, reflecting persistent digital divides as the older adults and less digitally literate individuals depended on assistance from younger family members or health workers. This highlights the dual responsibility of digital governance, that is, to broaden access while preventing digital exclusion. Building digital literacy at community level will be essential to ensure equitable empowerment (16).

### Data quality, interoperability, and system integration

While BVS data proved invaluable for planning and monitoring, fragmentation across digital platforms constrained efficiency. Health workers entered information separately into BVS, DHIS2, and other systems, increasing workload and the risk of inconsistencies. Similar challenges have been documented across LMICs, where donor-driven digital tools often operate in isolation (17, 18).

Bhutan’s Electronic Patient Information System (ePIS) aims to integrate multiple data streams including BVS, DHIS2, and Civil Registration and Vital Statistics (CRVS) using interoperability standards such as HL7 FHIR. This integration will reduce duplication and enhance data reliability (19, 20).

Linking BVS with the National Digital Identity (NDI) platform may further improve identity verification and reduce duplicate registration. Accurate denominator estimation is particularly challenging in Bhutan due to high internal mobility and seasonal migration patterns. Nomadic herders move between high mountain pastures in summer and lowlands in winter, often crossing district boundaries. Many citizens also travel frequently for pilgrimage, shopping, or official duties, and can access health services from any facility nationwide. In the era of paper-based records, such mobility made it difficult to calculate vaccination coverage accurately. Furthermore, the Population and Housing Census of Bhutan (PHCB) is conducted only once every 10 years, while district populations can fluctuate significantly when large projects commence or close. The introduction of unique registration numbers in the BVS, linked to citizens’ national identity (CID) numbers and phone numbers, substantially improved traceability, although duplication persisted when individuals forgot their identifiers.

Experiences from Estonia and Rwanda demonstrate that national digital identity systems can anchor health-data interoperability while safeguarding privacy (5, 21).

### Systemic capacity and implementation realities

Applying Potter and Brough’s systemic capacity-building framework (8) revealed constraints across performance, personal, workload, facility, and support capacities. Bhutan’s rapid digitalization outpaced development of the enabling environment for sustained use. Health workers reported inadequate hardware, unreliable internet, and limited technical support.

Addressing these challenges will require coordinated investments in user-friendly system design, district-level technical support, digital competencies among health workers, streamlined reporting processes, equitable access to devices and connectivity, and strengthened governance mechanisms for digital health oversight.

Bhutan’s experience underscores that digital transformation is as much institutional as technological. Health assistants responsible for service delivery often shoulder the entire data-entry burden. Without organizational reform to redistribute workload or automate reporting, digitalization may reinforce existing inefficiencies instead of alleviating them.

Another important operational insight concerned the sustainability and maintenance of digital equipment used for BVS implementation. Health workers reported that centrally procured laptops and desktops were often shared, inconsistently maintained, or became non-functional over time, contributing to interruptions in reporting and service delivery. Several participants suggested that providing a fixed digital equipment fund or ‘laptop quota’ directly to frontline health workers could improve ownership, maintenance, and continuity of use. Participants noted that such an approach might allow users to select devices suited to their operational needs, address procurement issues, and strengthen accountability for upkeep and functionality. Although exploratory, these perspectives highlight an important and under-discussed dimension of digital health implementation in LMICs: the long-term sustainability and usability of frontline digital infrastructure.

These reflections on equipment ownership parallel a broader need for professional ownership in digital health governance. Participants also highlighted the importance of involving health informatics experts, data analysts, and frontline users more meaningfully during system design and implementation. Such involvement may improve usability, analytical functionality, accountability, and long-term sustainability of digital health systems.

At policy level, the findings from the BVS study have informed revisions to the forthcoming National Digital Health Strategy 2025–2030, particularly regarding integration of ePIS, BVS, and NDI. Translating empirical evidence into strategic reform exemplifies the adaptive-governance cycle central to HRPS discourse.

### Comparisons and implications for LMICs

Few LMICs have implemented national vaccine registries that combine data integrity with citizen empowerment. Studies from Indonesia, Kenya, and Sri Lanka show that most systems prioritise logistics and reporting rather than governance transformation (2, 4, 12, 18, 22, 23). Similar integrated digital health approaches have also been reported from Rwanda and Guinea, where national digital platforms have supported interoperability, surveillance, and public-health coordination (5, 24). Bhutan’s model demonstrates that when community trust, political commitment, and public-sector ownership converge, both efficiency and citizen engagement can be strengthened (25).

Key lessons for other LMICs include the following:

- Contextual adaptation is critical: locally led implementation and stewardship of digital systems can enhance sustainability when aligned with global public goods and standards.
- Citizen-centred design may strengthen public engagement and acceptability of digital health systems: embedding user perspectives fosters uptake and accountability.
- Investment in digital infrastructure and human capacity is non-negotiable: sustainability depends on parallel growth in connectivity, skills, and institutions.

Consistent with the WHO Global Strategy on Digital Health 2020–2025 (22), Bhutan’s BVS illustrates learning-by-doing in a resource-limited setting. It shows how embedding digital platforms within governance systems may contribute to broader health-system strengthening and public participation goals.

### Study limitations

Several limitations should be acknowledged. First, qualitative data were collected from seven districts and may not represent all local contexts. Second, social desirability bias may have influenced responses given the government’s central role in BVS implementation. Finally, the reliance on descriptive analysis limits the ability to formally test associations, although integration with qualitative findings strengthens interpretability.

### Policy and practice implications

The BVS supported real-time monitoring, forecasting, and self-registration for vaccination which contributed towards achieving near-universal coverage during and after the COVID-19 pandemic. It enhanced accountability and transparency across system levels while granting citizens access to their own records.

Bhutan’s experience shows that locally developed, publicly governed digital systems, supported by social trust and political commitment, can strengthen both efficiency and participation in health governance, thereby offering actionable lessons for other LMICs pursuing digital-health reforms.

## Conclusion

This study demonstrates that digital platforms can serve as tools to support governance processes within resource-limited health systems, as illustrated by the Bhutan Vaccine System. By enabling live dashboard tracking, transparent reporting, and public access to information, BVS has strengthened the link between data, decision-making, and trust in public institutions.

Although challenges persist, particularly in interoperability, infrastructure limitations, digital literacy, and workload management, the high vaccination coverage achieved during the national programme underscores the potential of nationally owned digital innovations in LMICs.

Bhutan’s experience yields three key policy insights that may guide similar efforts elsewhere:

- Institutionalize interoperability across all health-data platforms through shared standards and digital-identity linkage.
- Invest in capacity building that simultaneously develops technical, managerial, and governance competencies.
- Embed citizen engagement throughout system design and implementation to ensure equity, trust, and sustainability.

As Bhutan advances its National Digital Health Strategy 2025–2030, the BVS provides a solid foundation for integrating digital governance across the broader health system. It offers useful lessons for other LMICs seeking to address health system challenges and improve citizen engagement through digital health interventions.

## Data Availability

The original contributions presented in the study are included in the article/Supplementary material, further inquiries can be directed to the corresponding author.
