# Supplementary material for: Enhancing health system governance and citizen empowerment in Bhutan through the Bhutan Vaccine System (BVS): a mixed-methods study
Source: Front Public Health. 2026 Jul 1;14:1828261. doi: 10.3389/fpubh.2026.1828261 (PMC13368996; doi:10.3389/fpubh.2026.1828261)
Supplement: Supplementary file 1 [file Supplementary_file_1.docx]

**Supplementary Annex 1: Key Question Guides**

A: Key Question Guides to Policy Makers (DHO, Programme Manager, HMIS Manager, NITAG, ICT, Policy Makers)

**Sir/Madam, thank you again for agreeing to participate in this research interview. Let me reiterate that I will be interviewing you as a researcher and not in my official capacity of being an employee of the Ministry of Health. Whatever you share with me shall be used solely for research purposes by the research team. Your personal identifiers shall not be used in the study findings dissemination nor shall be shared with anyone.**

**If my questions are not clear to you, then you could ask me to repeat them any number of times.**

1. Could you introduce yourself, please?

Probe: Please tell me your designation.

Say Thank You to the respondent

1. What do you think are the current main health system challenges in Bhutan?

How do you think we can address the main challenges highlighted above?

1. What is your opinion on the current digital interventions in Bhutan?

Prompt enablers, challenges, and bottlenecks

**Now we shall discuss your experience and opinion about the Bhutan Vaccine System (BVS) as a DHO/Programme Manager/HMIS Manager/Policy Maker.**

1. As a DHO/Programme Manager/HMIS Manager/Policy Maker, for what purposes do you use the BVS data/information at your level?

(Probe: Planning for vaccination, monitoring of vaccination, report generation)

4.1 Please share with us the challenges and the success stories experienced.

1. You know that for other routine vaccines, the health workers in Health Facilities maintain tallies, record them in the MCH register (most health facilities also enter the information in the DHIS2-based MCH Tracker System), compile them in the Monthly Activity Report, and enter them in the DHIS2. However, they enter the COVID-19, HPV, and Flu vaccine information directly on the BVS.

5.1 Do you still collect reports for those vaccines in BVS from the health facilities or do you generate them from the BVS?

5.2 What is the source of the denominator (target population) for coverage calculation?

5.3 How is the denominator (target population) at various levels (health facility, dzongkhag or national) determined?

5.4 Do you have guidelines or SOPs for coverage calculation?

1. Every health facility/Dzongkhag calculates vaccination coverage but for the routine vaccine (not currently included in the BVS) there is an issue of follow-up of individuals who avail of vaccination from other facilities.

6.1 Did the BVS address this issue (for those vaccines included in the BVS)?

1. In your opinion, what were the main political, policies, laws/legal/ethical, and organizational/health facilities factors that enabled the implementation and use of BVS?
2. In your opinion, what were the main political, policy, laws/legal/ethical, and organizational/health facilities factors that hindered the implementation and use of BVS?
3. What were the key lessons learned, while using the BVS?

Probe: Please share practical examples.

9.1 In your opinion, how do you think we can address/overcome these challenges?

1. In your opinion, how can we ensure the success & sustainability of Digital Health Interventions such as the Bhutan Vaccine System?
2. Before we conclude the interview, would you like to share anything else about the Bhutan Vaccine System or any other Digital Health Interventions that were not covered in our discussion?

**NOTE to the Interviewer: DO NOT FORGET TO THANK THE RESPONDENT**

**B: Key question guides to PHC managers or Health workers**

**Sir/Madam, thank you again for agreeing to participate in this research interview. Let me reiterate that I will be interviewing you as a researcher and not in my official capacity of being an employee of the Ministry of Health. Whatever you share with me shall be used solely for research purposes by the research team. Your personal identifiers shall not be used in the study findings dissemination nor shall be shared with anyone.**

**If my questions are not clear to you, then you could ask me to repeat them any number of times.**

1. Could you introduce yourself, please?

Probe: Please tell me your designation, number of years in service, and name of your health facility.

Say Thank You to the respondent

As a healthcare provider, you must have used the Bhutan Vaccine System (BVS). Now we shall discuss your experience and opinion about the BVS.

1. For which vaccines you have used the BVS?

(Note to interviewer: BVS has Covid-19, HPV, and Flu vaccine)

1. Can you describe the actual process of how the BVS was rolled out in your health facility?

Probe: training, information sharing, and workload.

3.1 Please share with us the challenges and the success stories experienced.

1. For other routine vaccines you maintain tallies, record them in the MCH register (most health facilities also enter the information in the DHIS2-based MCH Tracker System), compile them in the Monthly Activity Report, and enter them in the DHIS2. However, for COVID-19, HPV, and Flu vaccines you are supposed to directly enter information on the BVS on a real-time basis.

4.1 Do you still maintain paper-based records for those vaccines in BVS?

IF YES, why?

(Note to interviewer: Collect a sample/snapshot of the record)

4.2 Do you still have to submit reports for those vaccines in BVS?

(Note to interviewer: If YES, please collect a sample/snapshot of the report)

1. For what purposes do you use the BVS data/information at the health facility level?

Probe: Examples Health Facility Report (coverage calculation), tracking of unvaccinated individuals from your catchment area, future planning of vaccination stock (supply requisition/indents), etc

5.1 What is the source of the denominator (target population) for coverage calculation at your health facility?

5.2 Who determines the denominator (target population) at your facility?

5.3 Do you have guidelines or SOPs for coverage calculation at your health facility?

1. Every health facility calculates vaccination coverage but for the routine vaccine (not currently included in the BVS) there is an issue of follow-up of individuals who avail of vaccination from other facilities.

6.1 Did the BVS address this issue (for those vaccines included in the BVS)?

Probe: Explain

1. In your opinion, what were the main political, policies, laws/legal/ethical, and organizational/health facilities factors that enabled the use of BVS?
2. In your opinion, what were the main political, policy, laws/legal/ethical, and organizational/health facilities factors that hindered the use of BVS?
3. What were the key lessons learned, while using the BVS?

Probe: Please share practical examples.

- 1. In your opinion, how do you think we can address/overcome these challenges?

1. In your opinion, how can we ensure the success & sustainability of Digital Health Interventions such as the Bhutan Vaccine System?
2. Before we conclude the interview, would you like to share anything else about the Bhutan Vaccine System or any other Digital Health Interventions that were not covered in our discussion?

NOTE to the Interviewer: DO NOT FORGET TO THANK THE RESPONDENT

**C: Key Question guides to Community Members**

**Sir/Madam, thank you again for agreeing to participate in this research interview. Let me reiterate that I will be interviewing you as a researcher and not in my official capacity of being an employee of the Ministry of Health. Whatever you share with me shall be used solely for research purposes by the research team. Your personal identifiers shall not be used in the study findings dissemination nor shall be shared with anyone.**

**If my questions are not clear to you, then you could ask me to repeat them any number of times.**

1. Could you introduce yourself, please?

Probe: Please tell me your age and occupation.

(Note to interviewer: Say Thank You to the respondent)

Now we shall discuss your experience and opinion about the Bhutan Vaccine System (BVS) and/or the vaccines in the BVS.

1. Did you receive the Covid-19/HPV/Flu vaccine?

(Note to interviewer: These three vaccines are in the BVS)

2.1 IF YES, where did you go to get Covid-19/HPV/Flu vaccine?

Probe: The catchment area facility, or other health facility

2.2 IF YES, which document(s) did you carry while going to receive the vaccine (COVID-19/HPV/Flu vaccine)?

Probe: CID, Voter Card, SRP, Permit, Passport, MCH Card, etc or none

2.3 IF YES, could the healthcare provider easily find your information on the BVS?

(Note to interviewer: The Bhutanese population could go to any health facility in Bhutan for healthcare services including vaccination which are provided free of cost by the government.)

1. Did you register (or edit information) in the BVS yourself or did someone help you?

(Note to interviewer: The general public can register in the system themselves or anyone could register for them)

3.1 If themselves, please share your experiences and feedback, if any.

3.2 If not themselves, ask who registered them in the BVS.

1. Did you have adverse events or reactions following vaccination?

4.1 IF YES, did you experience these events or reactions after you were discharged from the 30-minute observation or during the observation period?

4.2 IF YES and after the 30-minute mandatory observation period, did you use the BVS to report the adverse event (reactions) following vaccination?

(Note to interviewer: The general public can report adverse events/AEFI using the BVS. If the events happened during the observation period, then the healthcare provider would have recorded it themselves on the BVS)

4.3 IF YES, please share your experiences and feedback, if any.

1. Did you use the BVS to view/download the vaccination records/certificates?

(Note to interviewer: The general public can view/download the vaccination records/certificates from the BVS)

5.1 IF YES, please share your experiences and feedback, if any.

1. All in all, as a vaccine recipient, did you feel empowered by the BVS in availing of the vaccination services?

Probe: Request them to elaborate with examples

(Note to interviewer: Patient Empowerment means a process in which patient understand their role, and are given the knowledge and skills to perform a task in an environment that recognizes community and cultural differences and encourages patient participation. On the BVS, people could read instructions, register for vaccination including consent to receive the vaccine (for themselves or others), view/edit/update details, and view/download vaccination records/certificates without having to visit health facilities.)

1. What do you think about the government’s policy and initiatives to digitalize the health system in Bhutan? (Talk about BVS and ePIS)

7.1 In your opinion, what are the challenges that community members face while availing of online health services in Bhutan?

7.2 In your opinion, how do you think we can address/overcome these challenges?

1. Before we conclude the interview, would you like to share anything else about the Bhutan Vaccine System or any other Digital Health Interventions that were not covered in our discussion?

NOTE to the Interviewer: DO NOT FORGET TO THANK THE RESPONDENTS
